# An in vitro study on the influence of laser-activated irrigation on infiltration and leakage of a dual curing-resin cement as an endodontic sealer

**DOI:** 10.2340/biid.v11.41098

**Published:** 2024-07-12

**Authors:** Laurine Marger, Marwa Abdelaziz, Enrico di Bella, Ivo Krejci, Clara Isabel Anton y Otero

**Affiliations:** Division of Cariology and Endodontology, CUMD – University Clinics of Dental Medicine, Faculty of Medicine, University of Geneva Geneva, Switzerland

**Keywords:** Composite resin, adhesion, sealing, microleakage, confocal microscopy

## Abstract

**Objectives:**

The study aims to investigate the effects of laser-activated irrigation on infiltration and microleakage of a dual-curing resin cement applied as a root canal sealer.

**Methods:**

Thirty-eight extracted upper molars were attributed to four experimental groups. Roots were mechanically enlarged and disinfected (NaOCl). Control samples (*n* = 11) were irrigated with conventional needles and three different lasers were used to activate the irrigant in the other groups (*n* = 9): 2.94 µm Er:YAG laser, 9,300 µm CO_2_ laser and 808 nm diode laser with a modified black coated laser tip. Final irrigation was performed in each group with ethylenediaminetetraacetic acid (EDTA), sodium hypochlorite (NaOCl) and sodium chloride (NaCl) activated with lasers and in the control without activation.

Dentin tubules were then labeled with a red fluorophore (Rhodamine B) and the root canals were sealed with a dual-curing resin cement (Paracore). The cement fixed the dye on the sealed and infiltrated dentin parts. To remove the Rhodamine not fixed by the cement, roots were then sectioned horizontally and immersed in hydrogen peroxide (H_2_O_2)_. The empty dentin tubules spaces were then labeled with a green fluorophore (Fluorescein) allowing the visualization of infiltration and microleakage by confocal microscopy.

**Results:**

Percentages of infiltration were significantly higher in the middle root third of the control and Er:YAG laser-activated samples compared to CO_2_ or diode laser groups. Microleakage was present in all experimental groups but significantly less after CO_2_ laser activation.

**Conclusion:**

Laser-activated irrigation impacted resin cement infiltration and microleakage dependent on the applied wavelength. Er:YAG laser activation led to higher values of infiltration and microleakage compared to CO_2_ and diode lasers.

## Introduction

Endodontic treatments aim primarily to prevent and/or eliminate apical periodontitis – an inflammatory tissue response to bacteria and their toxins, mainly located in the root tissues [1].

Apical periodontitis is often asymptomatic and only coincidentally detected as soon as follow-up radiographs are taken. The prevalence of apical periodontitis is recently described as 52% at an individual level and 39% for teeth that underwent an endodontic treatment [2].

Post-treatment apical periodontitis can be attributed to either an insufficient decrease of bacteria load in the root tissue and/or a reinfection due to leakage between the sealing/obturation material and the tooth – both incompatible with periradicular healing [3].

The complex anatomy of the root space with main and accessory canals and the root dentin tissue itself has challenged dentists since decades [3, 4].

Enhanced disinfection of the main root canal as well as lateral canals and dentin tubules contribute to improving long-term success rates of endodontic treatments [5]. This is due to the fact that the bacteria causing the infection can be found equally on the surfaces of the root canals and in dentinal tubules up to deep dentin layers and are difficult to eradicate [6].

The efficiency of passive irrigation with traditional syringes is highly dependent on the anatomic properties of the main root canal and the diameter and material of the irrigation needle. In straight and large root canal configurations superficial dentin layers in the apical region can be sufficiently disinfected. However, narrow anatomies and a so-called vapor lock – trapped air- may hinder the irrigant to clean and disinfect this region [7, 8].

Diverse methods to enhance disinfection via active irrigation, for instance, laser or ultrasonic activation of irrigants have shown promising results [5]. These tools can improve irrigant penetration in the apical part of the root and deeper into the dentin [9, 10].

Er:YAG laser activation of chelating agents such as EDTA was shown to clean the root canal surface and expose dentin tubule entrances independently on the root region [11, 12]. Literature reports that new laser wavelengths such as 9,300 µm CO_2_ laser and diode lasers can also enhance irrigant penetration into root dentin. Modified diode lasers were found to promote irrigant penetration in deep dentin layers [10]. Investigations on micromorphology of the root wall surface reported on melting patterns for both 9,300 nm CO_2_ and modified diode lasers [13].

Current endodontic protocols based on traditional syringe irrigation can efficiently remove bacteria in superficial root dentin layers. Potentially remaining bacteria in deep root dentin layers are kept at bay with an additional seal of the root dentin and an obturation of the main root canals. The obturation and sealing of the main root canal aims to prevent microleakage between the tooth and the restorative material [14]. A leaking seal potentially allows an undetectable passage of bacteria, fluids, molecules, or ions as nutrition supply and thus a re-infection of the root canal system [10]. Clinical studies show, that next to the disinfection, the quality of the root canal obturation in terms of the tightness of the seal and the working length directly influences the long-term success rates of the endodontic treatment [10].

Thus, both disinfection and sealing can be considered as two complementary factors that importantly contribute to the clinical success of endodontic treatments.

Conventional sealers based on epoxy resin that are until now considered as gold standard were shown to have biocompatibility issues and allow leakage as well as bacterial growth. Bioinert materials such as tricalcium silicate cement sealers are partially antimicrobial and have bioactivity, which might improve biological sealing of the root canal system. They have many ideal properties for sealing the root dentin but are reported to be limited due to potential solubility, dimensional instability as well as difficult retrievability [15]. This is why literature came up with the question of the necessity of a root canal obturation with conventional materials and methods [16–18].

A recent study investigating the possibility to infiltrate and seal the root dentin walls with a dual curing resin-cement originally applied for post cementation revealed promising results in terms of infiltration and decrease in microleakage [19].

This study aimed to investigate the influence of laser-activated irrigation with an Er:YAG laser, a 9,300 nm CO_2_ laser and a 808 nm diode laser on infiltration and leakage of a dual curing resin cement. The first null hypothesis stated that there is no difference in the infiltration of a resin-cement after laser-activated irrigation between experimental groups. The second null hypothesis stated no difference regarding microleakage between the experimental groups.

## Materials and methods

### Preparation and Laser activated irrigation

Sample size calculation was based on published results with an absolute difference of 18% of infiltration between the tested groups (mean infiltration resin-based cement in middle root sections 58.4%) [19]. With a power of 80% and a two-sided alpha error of 5% we calculated a required sample size of *n* = 9 roots per group.

Thirty-eight caries-free upper third molars with 11–12 mm long (distance from their apex to the CEJ), straight palatal roots were selected from a pool of anonymized extracted teeth from the surgical department of the University clinics of Geneva (HUG- Hopitaux Universitaires Genève, chirurgie maxillo-faciale et buccale). The local ethical committee considers pooled biobanks as irreversibly anonymized and waives the necessity for ethical approval. Teeth were stored after extraction at 6°C in a water-based solution of 0.02 g/mL thymol (Sigma-Aldrich, Steinheim, Germany). To localize palatal canal orifices, access to the pulpal chamber was created with a diamond bur (40 µm, Intensiv, Montagnola, Switzerland) in a highspeed (red) contra-angle. Apical size of the roots was controlled with a size 10 and 15 taper C-pilot file (K Files, MICRO-MEGA, Besançon Cedex, France) and diameters of larger than 15 were excluded from the study and replaced. After the visual determination of the working length (visual patency length minus 1 mm) with a K file 10, root canals were mechanically enlarged to a size of 40/0.04 (Hy Flex^TM^ EDM, Coltène/Whaldent GmbH, Langenau, Germany in a micromotor (CanalPro^TM^ Jeni, Coltène/Whaldent GmbH, Langenau, Germany)) with a crown down technique under constant rinsing with 3% NaOCl (2 mL of 3%NaOCl between each file, Hänseler Swiss Pharma, Herisau, Switzerland) with an open-ended endodontic needle (ENDO 30G, Transcodent, Kiel, Germany).

Subsequently, the root canals were subjected to a specific rinsing protocol (Table 1). To investigate the pure effects of laser activation, ultrapure water was applied with an open-ended endodontic needle (ENDO 30G, Transcodent, Kiel, Germany) as an irrigation liquid in the laser groups for both the mechanical preparation steps and for laser activation.

**Table 1:** Description of the experimental groups with applied laser parameters and final irrigation procedure.

Group	Device	Parameter	Irrigation procedure
Control	**Endodontic needle** (ENDO 30G, Transcodent, Kiel, Germany)	Inserted up to working length minus 1 mm	2 mL NaOCl after each filing step, 1 × 30 s laser activation EDTA (3 mL), 1 × 30 s laser activation distilled water (2 mL), 3 × 30 s laser activation NaOCl (3 mL), 1 × 30 s laser activation distilled water (2 mL)
1	**ER:YAG laser** (LiteTouch, Light Instruments, Israel; Tip (AS7075(×), 0.4 × 17 mm)	Ø 0.3 W (20 mJ, 15 Hz) Inserted in pulpal chamber	
2	**9.3 µm CO_2_ laser** (Solea, Convergent Dental, USA) (Ultra-guide handpiece with endo-tip Ø 1.25 mm)	Ø 0.4 W (40%, 14 Hz) Inserted in pulpal chamber	
3	**WISER 808 nm** (Wiser Doctor Smile 808 nm (Lambda SpA, Brendola, Italy) black coated tip Ø 0.2 mm;	Ø 0.4 W (1 W, 26,666 µs on, 40,000 off) Inserted up to working length minus 2 mm	

Teeth were attributed to four experimental groups. Final irrigation and laser activation were performed with EDTA (17%, pharma24 SA, Geneva, Switzerland), NaOCl (3%, Hänseler swiss pharma, Herisau, Switzerland) and distilled water for all four experimental groups as described in detail in Table 1. Irrigation in the control group (*n* = 11; published also in [19]) was performed with up- and down-movements with an 30G open-ended endodontic needle up to working length minus 1 mm in the laser groups (G1–G3) (*n* = 9); the liquid was injected into the root main canal with the same needle and the pulpal chamber was also filled. Er:YAG (Light Touch, Light Instruments Ltd., Yokneam, Israel) and CO2 laser (Solea, Convergent dental, Waltham, MA, USA) tips were activated at the root canal entrance while the tip of diode laser WISER (Lambda SpA, Brendola, Italy) was inserted up to the working length minus 2 mm and moved in coronal to apical direction in continuous helical movements at 1 mm/s. The diode laser tip was coated prior to use with carbon particles (Carbon black, acetylene 100%, compressed, 99.9+%, Alfa Aesar, Kandel, Germany) that were glued with a transparent glue-spray (Toolcraft, Conrad Electronic AG, Wollerau, Switzerland) [20]. During activation, the liquid level in the access cavity was constantly controlled and replenished when needed.

After activation, roots were dried subsequently with paper points (Hy Flex^TM^ EDM Paper Points, Coltène/Whaldent GmbH, Langenau, Germany).

To visualize infiltrated and sealed dentin as well as microleakage we followed a protocol designed to detect the material indirectly and avoid adding dyes to the sealers [19].

Prior to obturation, dentin was labeled with a red fluorophore. Samples were conserved for 24 h in an ethanolic solution of 0.1% RITC (Rhodamine B isothiocyanate, 283924-100MG, SIGMA-ALDRICH Chemistry, Steinheim, Germany).

### Root canal obturation and sample preparation for confocal microscopy analysis

Samples were then subsequently dried with paper points before sealing. Parabond Non-Rinse Conditioner (Coltène/Whaldent GmbH, Altstätten, Switzerland) was applied to all groups up to the working length for 30 s with a paper point, slightly air dried for 2 s. The canals were then sealed with a layer of Parabond adhesive that was mixed in a ratio of 1:1 (chemical cured adhesive A and B Coltène/Whaldent GmbH, Altstätten, Switzerland) and applied actively for 30 s in the root canal. Paper points soaked up excess bond and the canal was slightly airdried for 2 s. Paracore DENTIN SLOW (dual curing core & resin cement) (Coltène/Whaldent GmbH, Altstätten, Switzerland) was applied in a thin layer onto the canal walls and spread-out with a Gutta-percha point (Hy Flex^TM^ EDM Guttapercha Points, Coltène/Whaldent GmbH, Langenau, Germany) that was covered with Vaseline (Favodent Karl Huber, Karlsruhe, Germany) to facilitate its removal after the setting of the sealer.

Sealed teeth were stored at 37°C under 100% humidity for at least 7 days until sectioning. Two samples were obtained from each root at 2 and 4 mm from the apex (apical and middle root third) using Diamond Cut-off Wheel (M1D13, 127 mm dia. × 0.4 mm, Struers, Ballerup, Denmark) under constant water cooling. To visualize the infiltrated dentin area, obtained sections were then immersed in a 35% hydrogen peroxide solution (35%, Drogerie du Jura, Nyon, Switzerland) for 24 h to remove excess rhodamine not ‘fixed’ by the sealer.

After abundantly rinsing the sections in water for 60 s, they were immersed in a 50% ethanolic solution of 100 IM sodium fluorescein (Fluorescein sodium salt, 46970-100G-FSIGMA-ALDRICH Chemistry, Steinheim, Germany) for 3 min and washed in water for 10 s. This method allows differentiation between infiltrated and non-infiltrated or sealed dentin.

Prior to observation, root sections were fixed and mounted between slides and coverslips and protected from light.

### Confocal microscopy analysis

Middle and apical root sections were submitted to a confocal microscope (Zeiss Confocal Line scan LSM 800 Airyscan) at a 10-fold magnification and at a wavelength of λ_emission_ = 580 nm for Rhodamine B and λ_emission_ = 516 nm for Fluorescein. All sections were analyzed entirely based on four digitally assembled captures. As described previously in detail [19], the stack of the assembled images was corrected (Imaris 9.7.2; Andor Technology Limite, Belfast, Northern Ireland) and most appropriate frames selected (Matlab R2021a; MathWorks, Natick, Massachusetts, US).

Red and green fluorescence was then automatically quantified around the lumen in an area of 20 µm delimited manually using an image analysis software QuPath (Bankhead et al. QuPath: Open-source software for digital pathology image analysis. Scientific Reports (2017) as described in detail elsewhere [19]. The fluorescence calculation was based on the ratio of the number of red or green pixels and the number of pixels without fluorescence in the defined working area.

### Statistical analysis

All statistical analyses were run with Minitab 19.2020.1 (Minitab GmbH, State College, PA, US). Penetration and microleakage was evaluated using a two ways ANOVA test followed by Fisher’s LSD post-hoc test. Significance levels were set to *p* < 0.05.

## Results

### Sealer penetration

**Figure 1:**
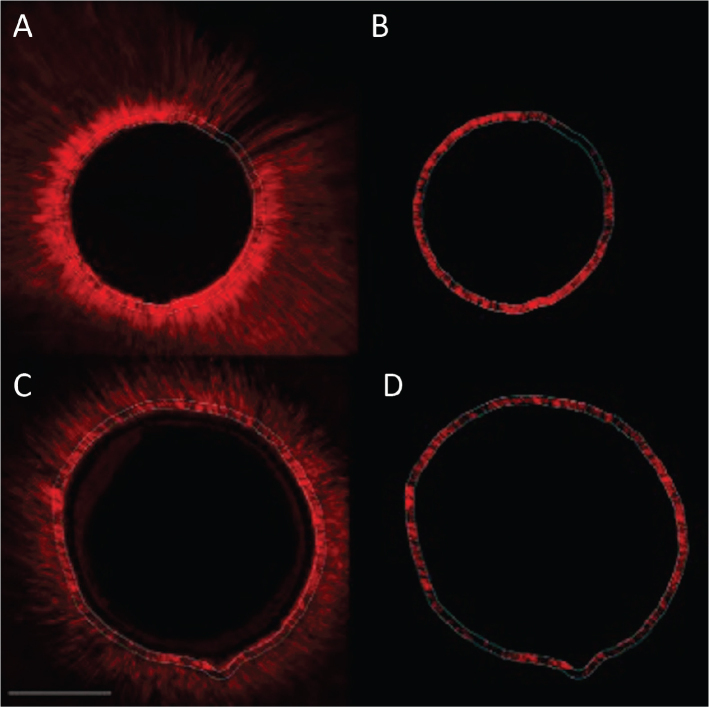
Confocal images with homogeneous sealer penetration in dentin tubuli for both, group 1 (Er:YAG) and 2 (CO_2_) in the apical root third. Group 1 (Er:YAG) (photos A and B) and group 2 (CO_2_) (photos C and D). Left images overview scan, right image with fluorescence for quantification in working area. Scale bar represents 200 µm.

The sealer penetration varied depending on the experimental group and the location in the root (middle or apical third) (see Figure 1).

The ANOVA test detected a statistically significant difference in penetration between the tooth region and the experimental groups with *p* = 0.001. Fisher’s post-hoc tests identified a statistically significant difference between the average values of the middle (61.62%) and the apical (44.46%) root thirds.

**Figure 2:**
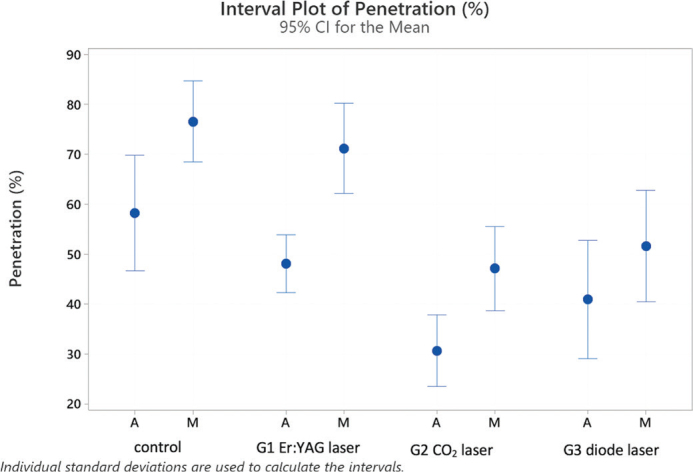
Interval plot with percentages of penetration of the sealer in laser groups 1–3 and the control group with traditional syringe irrigation, analyzed in middle (M) and apical (A) root sections.

Figure 2 provides the interval plots of the confidence intervals of means for the percentages of sealer penetration for the experimental groups and the control.

Concerning the experimental groups, Fisher’s post-hoc test identified two categories based on the penetration values: The control and G1 (Er:YAG laser) being equivalent with average values of 67.37% and 59.63% and G2 (CO_2_ laser) and G3 (diode laser) being equivalent with averages of 46.26% and 38.88% of penetration, respectively. These differences apply equally when the apical and middle sections are evaluated separately.

Considering the subgroups of Table 2, it is possible to identify four partly overlapping groups and two extreme subgroups: control M with the highest penetration level (76.5) and G2 (CO_2_ laser) A with the lowest level (30.65) (see Figures 1 and 2).

**Table 2:** Mean values for sealer penetration with standard deviation and grouping based on Fisher’s LSD test. Means that do not share a letter are significantly different.

Group	Mean (SD)	Grouping			
Control *Middle*	76.54 (11.3)	**A**			
G1 (Er:YAG) *Middle*	71.14 (11.8)	**A**	**B**		
Control *Apical*	58.20 (16.2)		**B**	**C**	
G3 (diode) *Middle*	51.61 (14.5)			**C**	
G1 (Er:YAG) *Apical*	48.12 (7.5)			**C**	**D**
G2 (CO_2_) *Middle*	47.11 (11.0)			**C**	**D**
G3 (diode) *Apical*	40.91 (15.3)			**C**	**D**
G2 (CO_2_) *Apical*	30.65 (9.3)				**D**

### Microleakage

**Figure 3:**
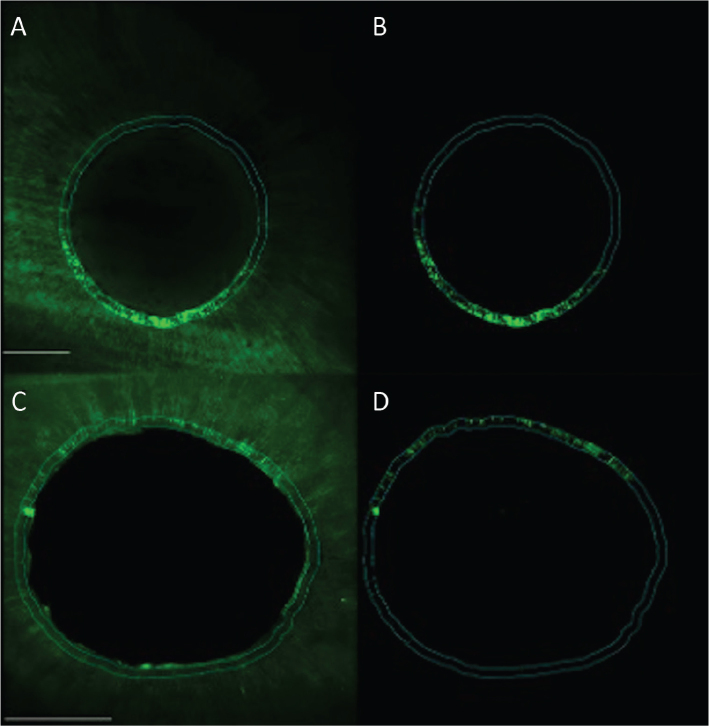
Confocal images representing the microleakage along the dentin tubules for experimental groups 1 (Er:YAG) and 2 (CO_2_) in the apical root third. Group 1 (Er:YAG) (photo A and B) and group 2 (CO_2_) (photo C and D). Left images overview scan and right image with fluorescence for quantification in working area. Scale bar represents 200 µm.

**Figure 4:**
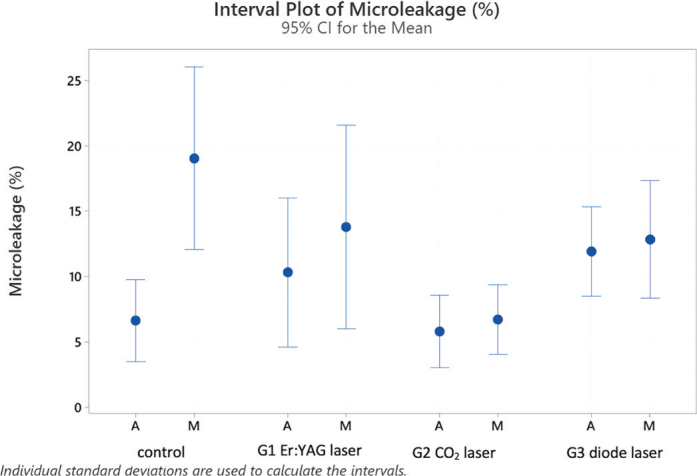
Interval plot with percentages of microleakage along the resin tags in laser groups 1–3 and the control group with traditional syringe irrigation, analyzed in middle (M) and apical (A) root sections.

Microleakage along the sealer tags was present in all experimental groups (see Figures 3 and 4). Figure 4 shows interval plots of the confidence intervals of means for the percentages of microleakage for all groups.

The ANOVA test detected a statistically significant difference in microleakage between tooth region (*p* ≤ 0.006) and the four groups (*p* = 0.017).

Fisher’s post-hoc tests identified a statistically significant difference between average values of the middle (13.19%) and the apical (8.55%) root thirds.

Concerning the experimental groups (average of apical and middle values combined), Fisher’s post-hoc test identified two clearly distinct categories: control, G3 (diode) and G1 (Er:YAG) being equivalent (average values of 12.83%, 12.38% and 12.04%) and G2 (CO_2_) with average of 6.24%.

Considering the subgroups represented in Table 3, it is possible to identify four partly overlapping groups and two extreme subgroups: highest microleakage levels in the control group in the middle root third (19.0%) and the lowest level in the apical section of the control group with (6.6%), G2 (CO_2_) in Middle (6.7%) and Apical (5.8%) sections.

**Table 3:** Mean values for microleakage with standard deviation and grouping based on Fisher’s LSD Test. Means that do not share a letter are significantly different.

Group	Mean (SD)	Grouping			
Control *Middle*	19.03 (9.7)	**A**			
G1 (Er:YAG) *Middle*	13.78 (10.1)	**A**	**B**		
G3 (diode) *Middle*	12.83 (5.8)		**B**	**C**	
G3 (diode) *Apical*	11.92 (4.4)		**B**	**C**	**D**
G1 (Er:YAG) *Apical*	10.30 (7.4)		**B**	**C**	**D**
G2 (CO_2_) *Middle*	6.69 (3.5)			**C**	**D**
Control *Apical*	6.63 (4.4)				**D**
G2 (CO_2_) *Apical*	5.79 (3.6)				**D**

If laser groups (G1–3) are merged together and compared to the control, there was no statistically significant difference (average: control [12.8%] and laser groups [10.22%]). However, regarding values noted separately at middle and apical sections, control values for microleakage are significantly higher than both G2 (CO_2_) and 3 (diode) in middle root sections but not in the apical region. G1(Er:YAG) is overlapping to both categories (Table 3).

## Discussion

This study aimed to investigate the influence of laser activated irrigation with an Er:YAG, a 9.3 µm CO_2_ and a 808 nm diode laser on the infiltration capacity of a resin-based cement in root dentin as well as the microleakage at the sealer-dentin interface.

The application of a resin-based cement in combination with a gutta-percha master point (control group) was investigated and compared to a conventional epoxy resin sealer elsewhere [19]. The protocol for the experimental resin group was elaborated considering the literature of root canal obstruction as mono-block with resin-based materials as Epiphany and Resilon that were considered as very promising in short-term results. However, in long-term studies, the results were less promising as the dentin–sealer interface was shown to leak due to gap formation [21, 22]. This can most probably be explained by important stress at the material–dentin interface based on the unfavorable C-factor of root canal configurations with high numbers of bonded and very few numbers of unbonded surfaces in root canals [23]. To avoid the described drawbacks of a mono-block formation of the sealer and the core material, the authors combined the resin cement with gutta-percha points that were impregnated with vaseline and served as a ‘stress breaker’ [24, 25]. This methodology allowed for a comparatively high infiltration of the resin into the dentin tissue combined with few microleakage [19].

Activation of irrigants with laser – that was done in this study prior to root canal sealing – could influence the seal dependent on the laser wavelength. The first null hypothesis of this study stating that infiltration of a resin-cement after laser activated irrigation would not be significantly different from the control group could only be accepted in part. It seemed that both CO_2_ and diode lasers reduced the sealer infiltration. In contrast, infiltration values after Er:YAG laser application and the control group were similar.

The absence of a difference in resin-cement infiltration in root dentin between the control and the Er:YAG laser group might be because EDTA irrigation with a 30G needle in a relatively large apical preparation of size ISO 40 was quite effective in opening dentin tubules entrances and that activation with Er:YAG laser did not have an additional effect on superficial root canal wall cleaning.

The application of a 9.3 µm CO_2_ and diode laser in EDTA led to significantly inferior values of resin penetration than the control and Er:YAG laser activation. This can be explained by the finding of our previous paper. We showed that LAI with a CO_2_ and diode laser in water leads to important heating of the irrigant and melting of the dentin. The melted dentin obstructs and seals thus the tubule entrances [13, 26].

Melting patterns might be explained by the laser wavelengths. A 9,300 nm CO_2_ laser is better absorbed by hydroxyapatite than water, while 808 nm diode lasers are not absorbed by water. However, it was shown that a black coating of the diode laser tip enabled the laser to work as a ‘hot-tip’ and to introduce vapor bubble formation in transparent irrigants. The heat from the laser tip can hereby lead to melting of the superficial dentin [20].

Resin penetration was impacted both by the application of laser activation of irrigants and the root dentin anatomy. The infiltration values were higher in middle than in apical root sections in all experimental groups. This might be explained by the decrease in the number of dentin tubules from coronal to apical and by the impact of physiological sclerosis starting from the root apex [27].

Similar to the first null hypothesis, the second hypothesis stating no difference in regard to microleakage between the experimental groups could only be partially accepted. Microleakage values in the control and Er:YAG laser groups were similar and overlapping with diode lasers. The CO_2_ laser group revealed significantly less microleakage than the control and Er:YAG laser groups. It seemed thus that the superficial melting of the root dentin walls had a positive impact on the prevention of microleakage. Dentin sealed root walls might be less prone to microleakage than resin infiltrated and sealed tissues. This is even more interesting when comparing to microleakage values from conventional epoxy resin sealers. We found in a previous study a microleakage value of about 35 in the middle root third compared to resin sealer infiltration with 11 and 7 in combination with CO_2_ laser application [19].

It is interesting to relate the findings of this study to results from clinical studies on endodontic treatment success in relation to patients age and root dentin sclerosis.

It was assumed that age related physiological sclerosis could positively impact endodontic treatment outcome by preventing bacteria from colonizing dentin tubules with reduced diameters [28, 29]. We might therefore raise the question if a ‘natural’ sealing of the dentin walls with laser melted superficial dentin would be more efficient than a conventional sealing with sealers.

It would be important to investigate the microleakage’s origin in future research. The results of this study do not consider this aspect as the sections were immersed in the dye and it is possible that the dye penetrated starting from four different surfaces: the main root canal, the external root surface as well as the both surfaces from cutting the sections. It might be possible that the microleakage values were overestimated. Moreover, the impact of an obstruction with a gutta-percha filling of the main root canal should be investigated as this has been removed in our study prior to cutting the sections. The gutta-percha obstruction was removed in order to avoid any monobloc formation and image acquisition issues with the confocal microscopy due to its important fluorescence.

Laser activated irrigation is reported to be less dependent on the preparation size of the main root canals than conventional needle irrigation [30]. This is why it would be interesting to test the lasers also in smaller preparation sizes and to investigate the possibility of new laser tip designs that might contribute to a favorable energy distribution and a more homogeneous surface pattern of the dentin.

## Conclusion

Within the limitations of the study, it can be concluded that laser activated irrigation can impact sealer infiltration in root dentin and microleakage dependent on the laser wavelength. The control group without laser application led to the highest infiltration and microleakage values. Er:YAG laser activation led to higher values of infiltration and microleakage compared to CO_2_ and diode laser application. It could be questioned if laser application led to a better seal compared to the control.

## References

[1] Kakehashi S, Stanley HR, Fitzgerald RJ. The effects of surgical exposures of dental pulps in germ-free and conventional laboratory rats. Oral Surg Oral Med Oral Pathol. 1965;20:340–9. 10.1016/0030-4220(65)90166-0 14342926

[2] Tibúrcio-Machado CS, et al. The global prevalence of apical periodontitis: a systematic review and meta-analysis. Int Endod J. 2021;54(5):712–35. 10.1111/iej.13467 33378579

[3] Siqueira JF, Jr. Aetiology of root canal treatment failure: why well-treated teeth can fail. Int Endod J. 2001;34(1):1–10. 10.1046/j.1365-2591.2001.00396.x 11307374

[4] Tabassum S, Khan FR. Failure of endodontic treatment: the usual suspects. Eur J Dent. 2016;10(1):144–7. 10.4103/1305-7456.175682 27011754 PMC4784145

[5] Verma A, et al. A randomized controlled trial of endodontic treatment using ultrasonic irrigation and laser activated irrigation to evaluate healing in chronic apical periodontitis. J Clin Exp Dent. 2020;12(9):e821–9. 10.4317/jced.56368 32994870 PMC7511050

[6] Ando N, Hoshino E. Predominant obligate anaerobes invading the deep layers of root canal dentin. Int Endod J. 1990;23(1):20–7. 10.1111/j.1365-2591.1990.tb00798.x 2391177

[7] Agarwal A, et al., Evaluation of apical capor lock formation and comparative evaluation of its elimination using three different techniques: an in vitro Study. J Contemp Dent Pract. 2017;18(9):790–4. 10.5005/jp-journals-10024-2128 28874643

[8] Tay FR, et al. Effect of vapor lock on root canal debridement by using a side-vented needle for positive-pressure irrigant delivery. J Endod. 2010;36(4):745–50. 10.1016/j.joen.2009.11.022 20307757 PMC2844877

[9] Susila A, Minu J. Activated irrigation vs. conventional non-activated irrigation in endodontics – a systematic review. Eur Endod J. 2019;4(3):96–110. 10.14744/eej.2019.80774 32161895 PMC7006592

[10] Anton y Otero CI, Marger L, di Bella E, Feilzer A, Krejci I, Abdelaziz M. An in-vitro study on effects of laser activation on dye penetration in human root dentin. Biomater Investig Dent. 2024;11:40311. 10.2340/biid.v11.40311 PMC11022650 38645926

[11] Zhu X, et al. Comparison of the antibacterial effect and smear layer removal using photon-initiated photoacoustic streaming aided irrigation versus a conventional irrigation in single-rooted canals: an in vitro study. Photomed Laser Surg. 2013;31(8):371–7. 10.1089/pho.2013.3515 23863104 PMC3732410

[12] Mancini M, et al. FESEM evaluation of smear layer removal from conservatively shaped canals: laser activated irrigation (PIPS and SWEEPS) compared to sonic and passive ultrasonic activation-an ex vivo study. BMC Oral Health. 2021;21(1):81. 10.1186/s12903-021-01427-0 33618701 PMC7901090

[13] Anton y Otero CI, et al. Micromorphology of root canal walls after laser activated irrigation. Eur J Prosthodont Restorat Dent. 2023;32(1):109–19. 10.1922/EJPRD_2600AntonyOtero11 37988616

[14] Duncan HF, et al. Treatment of pulpal and apical disease: the European Society of Endodontology (ESE) S3-level clinical practice guideline. Int Endodont J. 2023;56(S3):238–95. 10.1111/iej.13974 37772327

[15] Aminoshariae A, Primus C, Kulild JC. Tricalcium silicate cement sealers: do the potential benefits of bioactivity justify the drawbacks? J Am Dent Assoc. 2022;153(8):750–60. 10.1016/j.adaj.2022.01.004 35260235

[16] Roggendorf MJ, et al. Influence of moisture on the apical seal of root canal fillings with five different types of sealer. J Endod. 2007;33(1):31–3. 10.1016/j.joen.2006.07.006 17185125

[17] Burkovski A, Karl M. Lack of evidence for the necessity of root canal obturation. Quintessence Int, 2019;50(1):22–8. 30411092 10.3290/j.qi.a41335

[18] Sabeti MA, et al. Healing of apical periodontitis after endodontic treatment with and without obturation in dogs. J Endodont. 2006;32(7):628–33. 10.1016/j.joen.2005.12.014 16793468

[19] Anton y Otero CI, et al. Evaluating the use of self-conditioning adhesive combined with dual curing resin cement as an endodontic sealer: an in vitro study. Biomater Investig Dent. 2023;10(1):2282523. 10.1080/26415275.2023.2282523 PMC11229672 38979096

[20] Anton Y Otero CI, et al. Activation of endodontic irrigants using a 9.3 µm CO_2_ and diode lasers: A laboratory proof of concept model. Am J Dent. 2024;37(1):39-46. PMID: 38458982. 10.3389/fdmed.2022.1010916 38458982

[21] Pirani C, et al. Does hybridization of intraradicular dentin really improve fiber post retention in endodontically treated teeth? J Endodont. 2005;31(12):891–4. 10.1097/01.don.0000164853.92310.e7 16306825

[22] Ekambaram M, Yiu CKY, Matinlinna JP. Bonding of adhesive resin to intraradicular dentine: a review of the literature. Int J Adhesion Adhesives. 2015;60:92–103. 10.1016/j.ijadhadh.2015.04.003

[23] Feilzer AJ, De Gee AJ, Davidson C. Setting stress in composite resin in relation to configuration of the restoration. J Dent Res. 1987;66(11):1636–9. 10.1177/00220345870660110601 10872397

[24] Bouillaguet S, et al. Microtensile bond strength between adhesive cements and root canal dentin. Dent Mater. 2003;19(3):199–205. 10.1016/S0109-5641(02)00030-1 12628431

[25] Tay FR, et al. Geometric factors affecting dentin bonding in root canals: a theoretical modeling approach. J Endodont. 2005;31(8):584–9. 10.1097/01.don.0000168891.23486.de 16044041

[26] Anton y Otero CI, et al. Activation of endodontic irrigants using a 9300nm CO2 and diode lasers: am in-vitro proof of concept model. Am J Dent. Under review. 38458982

[27] Carrigan PJ, et al. A scanning electron microscopic evaluation of human dentinal tubules according to age and location. J Endod. 1984;10(8):359–63. 10.1016/S0099-2399(84)80155-7 6590745

[28] Ricucci D, et al. A prospective cohort study of endodontic treatments of 1,369 root canals: results after 5 years. Oral Surg Oral Med Oral Pathol Oral Radiol Endod. 2011;112(6):825–42. 10.1016/j.tripleo.2011.08.003 22099859

[29] Fouad AF, Burleson J. The effect of diabetes mellitus on endodontic treatment outcome: data from an electronic patient record. J Am Dent Assoc. 2003;134(1):43–51; quiz 117–8. 10.14219/jada.archive.2003.0016 12555956

[30] Wen C, et al. Effectiveness of photon-initiated photoacoustic streaming in root canal models with different diameters or tapers. BMC Oral Health. 2021;21(1):307. 10.1186/s12903-021-01671-4 PMC8207708 34130673

